# Supplementary material for: Multicenter Study of Benign Thyroid Nodules with Radiofrequency Ablation: Results of 762 Cases over 4 Years in Taiwan
Source: J Pers Med. 2022 Jan 6;12(1):63. doi: 10.3390/jpm12010063 (PMC8782025; doi:10.3390/jpm12010063)
Supplement: Supplementary file 1 [file jpm-12-00063-s001.zip › jpm-1522673-supplementary.pdf]

Table S1. Demographic data of 5 Participating Medical Centers.

Hospitals and Department	No. of Patients	No. of Nodules	No. of Sessions	Small (≤10mL)	Medium (11-30mL)	Large (>30mL)
Department of Diagnostic Radiology, Kaohsiung Chang Gung Memorial Hospital	376	435	439	180	135	120
Department of Medical Imaging, Chung Shan Medical University Hospital	122	122	122	51	59	12
Department of Surgery, National Taiwan University Hospital	100	100	100	45	37	18
Department of Otolaryngology, Cathay General Hospital	102	102	102	60	31	11
Department of Surgery, MacKay Memorial Hospital	62	67	68	15	33	19
Combined hospitals	762	826	831	351	295	180

Table S2. Volume reduction ratio (VRR) by different specialists.

VRR (%)	Radiologist	Otolaryngologist	Surgeon
Total			
1-month	38.45 ± 56.22	27.53 ± 10.27	41.23 ± 26.64
3-month	61.68 ± 26.34	54.32 ± 19.97	54.05 ± 25.31
6-month	73.42 ± 23.39	73.21 ± 18.88	57.78 ± 28.12
Small			
1-month	36.20 ± 39.62	26.91 ± 10.99	35.40 ± 33.36
3-month	62.26 ± 24.80	52.87 ± 21.44	48.31 ± 30.65
6-month	76.32 ± 17.15	71.29 ± 21.25	60.70 ± 34.54
Medium			
1-month	42.03 ± 22.06	30.69 ± 8.72	43.64 ± 24.83
3-month	60.96 ± 16.47	60.59 ± 16.82	60.25 ± 18.47
6-month	70.71 ± 16.22	78.28 ± 12.15	58.81 ± 22.31
Large			
1-month	49.84 ± 19.06	22.99 ± 7.62	39.08 ± 28.70
3-month	65.20 ± 14.65	46.62 ± 15.05	49.41 ± 28.80
6-month	75.48 ± 11.01	70.99 ± 17.28	56.90 ± 32.68
